# Effect of Workpiece/Tool Heat Transfer and Friction Coefficients on Accuracy of Simulated Temperatures and Torques in a Friction Stir Welding Plunge

**DOI:** 10.3390/ma17010198

**Published:** 2023-12-29

**Authors:** Matthew Goodson, Ryan Melander, Michael P. Miles, Troy Munro

**Affiliations:** 1 Mechanical Engineering Department, Brigham Young University, Provo, UT 84602, USA; goodson2@byu.edu (M.G.); ryanm55@student.byu.edu (R.M.); troy.munro@byu.edu (T.M.); 2 Manufacturing Engineering Department, Brigham Young University, Provo, UT 84602, USA

**Keywords:** finite element models, friction stir welding, workpiece/tool interfacial heat transfer coefficient, friction coefficient

## Abstract

Friction stir process models are typically validated by tuning heat transfer and friction coefficients until measured temperatures in either the tool or workpiece, but rarely in both, match simulated results. A three-dimensional finite element model for a tool plunge in an AA 6061-T6 is validated for temperature predictions in both the tool and workpiece using a friction coefficient that varies with time. Peak workpiece temperatures were within 1.5% of experimental temperatures and tool temperatures were off by 80 °C. The sensitivity of the predicted temperatures with respect to the workpiece/tool heat transfer coefficient was shown to be high for the tool and low for the workpiece, while the spindle torque was slightly underpredicted in the best case. These results show that workpiece/tool interface properties must be tuned by considering predictions on both sides of the heat generation interface in order to ensure a reliable process simulation.

## 1. Introduction

**Figure 1:**
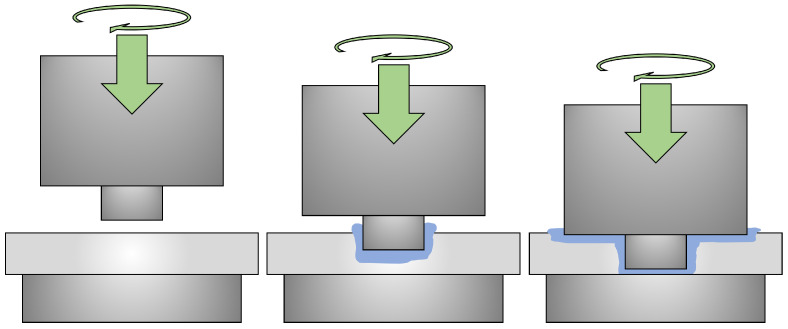
Schematic showing the plunge step of FSW, where the pin engages the workpiece, and then the shoulder is plunged into the material.

Friction stir welding (FSW) is a solid-state joining process with advantages over common fusion welding approaches. The process involves a rapidly rotating tool with a pin that is plunged into the parts to be welded, a dwell phase to increase heat, and then a traverse phase along the joint to create a weld. The plunge process is shown in Figure 1 and exhibits many of the extreme thermomechanical processes that are characteristic of FSW. Since the welding temperatures remain below the melting point of the material, the physical properties of the weld are often better than those associated with fusion welding [1]. FSW has been used to weld aluminum, copper, and dissimilar metals, which are typically unweldable using fusion-based processes [2]. With these advantages, FSW has seen widespread adoption in the automotive, aerospace, and rail transportation industries [3].

Since the invention of FSW in 1991 [4], experimental efforts have developed the process into a viable approach for many applications, but these development efforts are typically trial-and-error-based. Models of the FSW process began to appear in the early 2000s [5,6,7,8,9,10,11,12] in an effort to better understand the physics of the process and to speed up its development for industrial applications. These models are typically validated against experimental data, with various levels of rigor. However, the model inputs are not always measured independently from the model development and are simply adjusted to align simulation predictions with experiments.

The two most cited papers in the FSW heat transfer modeling space are from Chao and Khandkar, both of whom used thermocouples for validation measurements. Chao et al. [6] employed nine thermocouples in an AA 2195 workpiece at different distances from the weld center-line and five thermocouples attached to an M2 steel tool at varied distances above the shoulder. Commercial finite element codes, ABAQUS and WELDSIM, were used to model the steady-state heat transfer of the tool and the transient heat transfer of the workpiece, respectively. A good match was made between experimental and simulation temperature profiles by fitting the heat input to the workpiece and to the tool. The tool and workpiece were modeled separately, and in each case, the heat input was the fitting variable but with no reference to a physical law. Khandkar et al. [9] also matched an experiment with a model, where a moving heat input was used as a boundary condition. A total of 25 thermocouples were embedded in an AA 6061-T651 workpiece to measure temperature during the experiment. Good agreement was found between the experiment and model predictions. The heat transfer coefficient at the workpiece/backing plate interface ($h_{W/B}$) was varied to study its effect on the results and to find a good match. A value of $h_{W/B}$ = 1000 W m${}^{-2}$ K${}^{-1}$ provided the best result. The tool and the workpiece were modeled, but validation of the model temperatures was only performed on the workpiece. The model relates heat generation to physical laws, which is an improvement on previous models.

Temperature measurements in the workpiece are the most common method to validate models [3,13,14,15,16,17,18]. Andrade et al. [19] fitted model workpiece temperature profiles to hundreds of experiments conducted on aluminum to determine trends in the torque and workpiece temperatures based on the geometry and welding parameter inputs. Fewer papers have used temperature measurements in the tool to validate models [2]. Nakamura et al. [20] focused on matching simulation tool temperatures to experiments and found that $h_{W/B}$ = 2000 W m${}^{-2}$ K${}^{-1}$ provided the best agreement with an experiment using an AA 6061-T6 workpiece on an undefined backing plate. Danesh et al. [21] validated a model using both tool and workpiece temperature measurements, but they provide no information on the interfacial condition between the tool and workpiece other than defining the heat generation.

**Figure 2:**
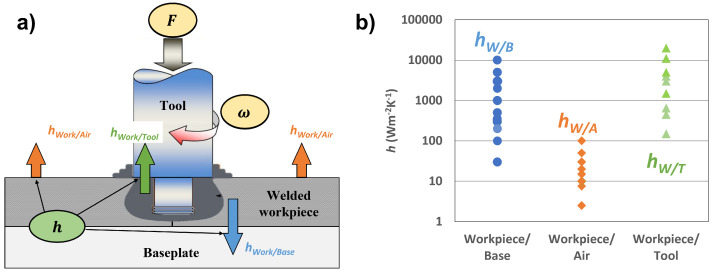
(**a**) Interfaces in an FSW model whereby the different *h* values must be specified; (**b**) the range of heat transfer coefficient values that have been used over the last 20 years [23]. Copyright 2021, Springer Nature.

Accurately defining boundary conditions is important for having a robust model. The three heat transfer interactions when modeling FSW are the following: the heat transfer between the tool or workpiece and the environment ($h_{W/A}$), the heat transfer between the workpiece and the backing plate ($h_{W/B}$), and the heat transfer between the workpiece and the tool ($h_{W/T}$). Figure 2 shows the variation in the literature for these three heat transfer coefficients. Of the three, the heat transfer coefficient at the workpiece/tool interface, $h_{W/T}$, has not been measured directly via experimentation and is thus a parameter that is adjusted to tune model results [22]. If a measurement of $h_{W/T}$ could be conducted, then the only remaining fitting parameter would be the friction coefficient. However, the sensitivity of workpiece and tool temperatures to variations in $h_{W/T}$ needs to be understood prior to designing such experiments.

Despite the models and simulations that have been developed and the advances in accuracy and understanding of the physics behind FSW, we have not seen models in the literature that were validated simultaneously in both the workpiece and the tool. This calls into question whether the heat generation at the workpiece/tool interface was accurately simulated, as it is fairly straightforward to tune a model in order to match temperatures for one side of the interface. This paper presents a model of the plunge phase of FSW where both the tool and workpiece temperatures were modeled and validated through experimentation. The effect of varying the heat transfer coefficient between the workpiece and the tool, $h_{W/T}$, on the predicted temperatures will be discussed. The role of the friction levels in achieving a good agreement between the simulation and experiment will also be considered.

## 2. Methods

### 2.1. Experimental Procedure

**Figure 3:**
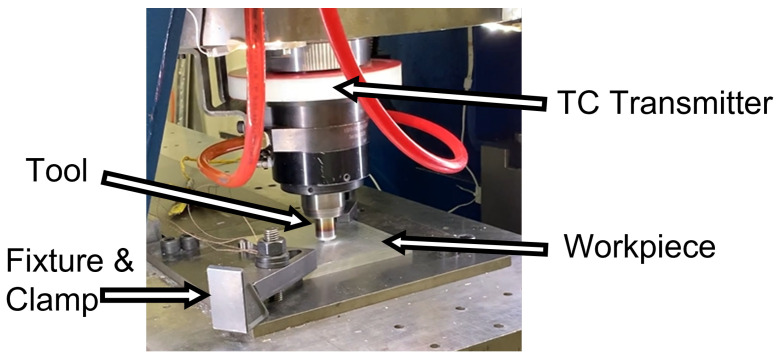
FSW plunge experiment system, including TC instrumentation and fixtures.

The plunge phase of FSW was carried out experimentally on AA 6061-T6 plates using a tool made of H13 steel. The machine used for the experiments is a TTI High Stiffness RM2 FSW machine (Bond Technologies, Elkhart, IN, USA). A Bond Technologies B&R-based programmable logic controller with high-speed data acquisition and control was used to program the welding parameters [24]. The machine controls rotation speed, tool displacement, and force. The FSW machine holds the tool and a Bluetooth collar for relaying thermocouple data [25]; see Figure 3. Thermocouple data were recorded in the tool and workpiece; for full experiment description see, Table S1 and Figure S1 in Section S1.1.

### 2.2. Numerical Modeling

**Figure 4:**
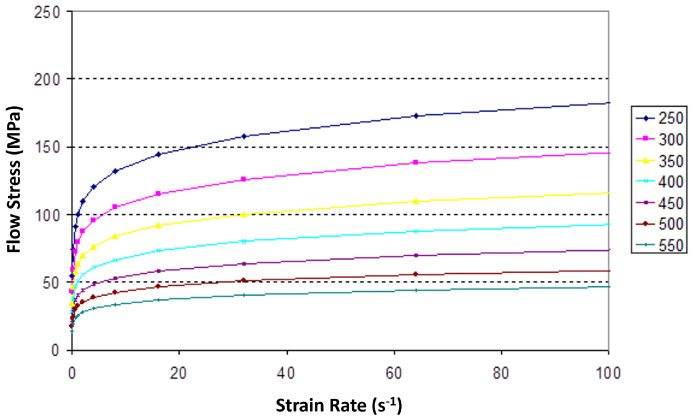
Temperature-dependent flow stress values for aluminum used in the model; temperature in °C.

The model was developed using the ForgeNxt software v.3.2 [26], which has the ability to simulate large strain and thermomechanical processes. An isotropic, viscoplastic Norton–Hoff law was used to model the evolution of material flow stress as a function of strain, strain rate, and temperature, see Figure 4. The expression for the deviatoric stress tensor, *s*, is shown below:

(1) $$s=2K{\left(\sqrt{3}\dot{\bar{\epsilon }}\right)}^{m-1}\dot{\epsilon }$$

where $\dot{\epsilon }$ is the strain rate tensor, $\dot{\bar{\epsilon }}$ is the effective strain rate, *K* is the material consistency, and *m* is the strain rate sensitivity. *K* (Equation (2)) is a function of temperature *T* and equivalent strain $\bar{\epsilon }$, where *n* is the strain hardening exponent, β is a thermal softening parameter, and $\epsilon_{0}$ is the prestrain term:

(2) $$K=K_{0}{\left(\epsilon_{0}+\bar{\epsilon }\right)}^{n}e^{\frac{\beta }{T}}$$

This viscoplastic law is capable of modeling material flow stresses in the region of the weld while providing the contact stresses with the tool that are used to calculate the friction shear stress at the workpiece/tool interface.

Friction at the workpiece/tool interface was modeled using Norton’s viscoplastic law, which simulates the shearing of a boundary layer of workpiece material in order to calculate the shear stress at the workpiece/tool interface:

(3) $$\tau \left(v\right)=-\alpha K|\Delta v_{s}{|}^{p_{f}-1}\Delta v_{s}$$

where α is the viscoplastic friction coefficient, *K* has been seen previously in Equation (2), $\Delta v_{s}$ is the relative sliding velocity at the workpiece/tool interface, and $p_{f}$ is the sensitivity to sliding velocity, which is equivalent to the strain rate sensitivity for the workpiece material [27].

Heat generated by plastic deformation is modeled according to the following term:

(4) $${\dot{q}}_{v}=f\bar{\sigma }\dot{\bar{\epsilon }}$$

where $\bar{\sigma }=\sqrt{\frac{3}{2}s:s}$ is the equivalent stress, and the factor *f* takes into account the fraction of energy converted into heat, which was taken as 0.9 in this paper [28]. Heat generation from friction at the workpiece/tool interface is given by the following:

(5) $${\dot{q}}_{f}=\tau \cdot \Delta v_{s}$$

where τ is the friction shear stress given by Equation (3). Frictional heat is shared between the workpiece and tool as a function of the effusivities of each, where the material with higher effusivity receives a greater proportion of the frictional heat. Effusivity is defined as $\sqrt{\rho ck}$, where ρ is density, *c* is heat capacity, and *k* is thermal conductivity; for further model description, see Figures S2–S4 and Table S2 in Section S1.2.

### 2.3. FSW Plunge Model

**Figure 5:**
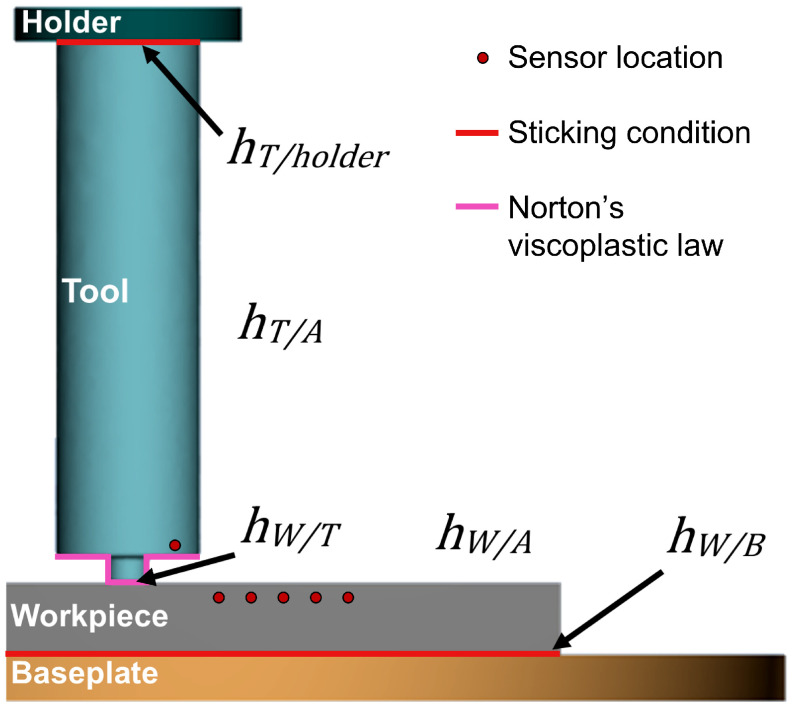
Schematic detailing the location of the different thermal boundary conditions and frictional boundary conditions. The baseplate and holder were set at a constant temperature of 20 °C.

Boundary conditions and sensor locations were set to complete the model. The various values for the heat transfer coefficients and friction coefficients were referenced from the literature values or determined by tuning of the model. Figure 5 shows the sensor locations and boundary conditions with the following parameters:

1. $h_{W/T}$ = 10, 20, 30, 40, and 50 kW m${}^{-2}$ K${}^{-1}$ (40 kW m${}^{-2}$ K${}^{-1}$ used for temperature matching);
2. $h_{T/A}=h_{W/A}=10$ W m${}^{-2}$ K${}^{-1}$ [20];
3. $h_{W/B}=500$ W m${}^{-2}$ K${}^{-1}$ [15];
4. α= s curve defined according to Norton’s viscoplastic law (see Figure S6);
5. $h_{T/holder}=$ adiabatic;
6. $T_{\infty }=28$ °C.

Some simplifications were made with the geometry and boundary conditions between the tool and the tool holder; for full justification, see Section S1.3.

The tool was meshed with 116,339 elements and the workpiece was meshed with 49,547 elements, with increased mesh density in regions expected to experience high strain rate and temperature gradients. Given the high strains in the portions of the workpiece under the tool, zones were defined for remeshing, which was necessary to avoid element distortion. High mesh densities were maintained in these zones, thereby capturing the high strain rate and temperature gradients inherent in the process.

## 3. Analysis/Results

### 3.1. 3D Model Temperature and Torque Validation

Validation of the 3D model was accomplished by comparing the temperature data at the same locations as the thermocouples in an experiment. In addition to the thermal histories in the workpiece, the tool temperature was also recorded, along with the tool’s vertical displacement and both the spindle loads and torques. Difficulty arose in matching all these parameters accurately because of the highly coupled processes involved in the FSW process. For example, there is not a one-to-one relationship between changing an input parameter and the output. Previous models in the literature have dealt with these difficulties by limiting model fitting based solely on either the tool or workpiece temperature [9,13,14,15,20]. The current work has attempted to develop a model that can predict all these parameters simultaneously compared to previous work, where these effects are often studied separately.

**Figure 6:**
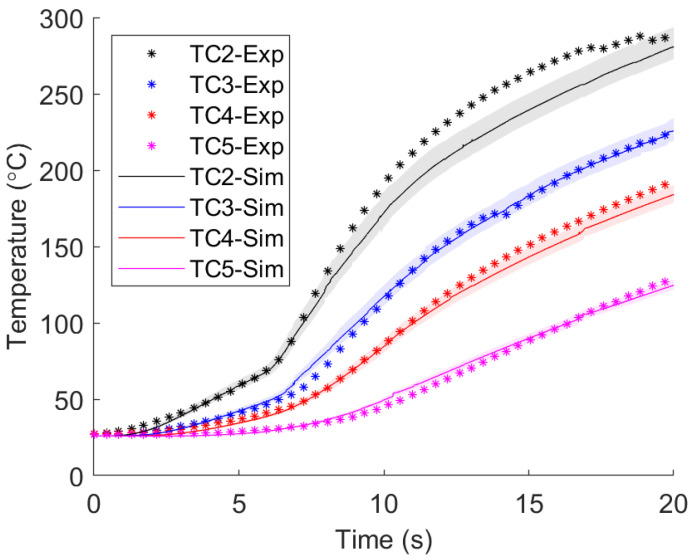
Resulting temperatures within the workpiece after tuning of the friction curve (see, Figure S6). Markers represent measurements at each position, and simulation data are represented by a solid line with a shaded error region around the line that accounts for possible error in thermocouple positioning.

Figure 6 shows the result of tuning the friction curve such that the simulated temperatures match the experimental thermocouple data. A region of error due to thermocouple positioning that overlapped many of the experimental markers was included, meaning that the simulated data were within the calculated error of the experimental data, (see Section S2 for the fitting process and Figure S6 for the TC error explanation). Also, the percent differences between the peak experimental temperatures and peak simulated temperatures were less than 1.5% for all sensor locations.

**Figure 7:**
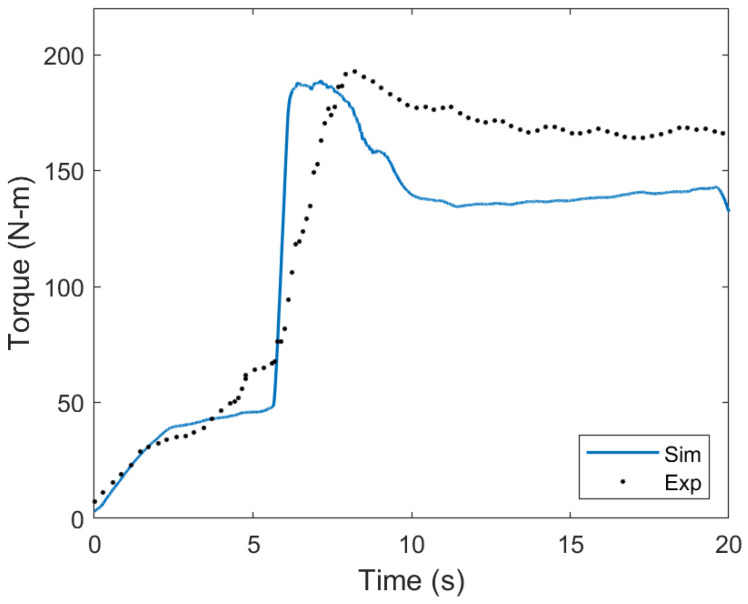
Simulated and experimental torque values from FSW plunge. The simulated values were smoothed with a running average.

The torque from the simulation was calculated and compared to experimental values to validate mechanical performance. The following equation was used to estimate the simulation torque:

(6) $$\begin{array}{cc} P & =T\omega \\ P & =\frac{\pi }{30}TN_{rpm} \end{array}$$

where *P* is the power in watts, *T* is the torque in N-m, and ω is the rotational velocity in rad s${}^{-1}$. Equation (6) was simplified for rotational velocity in rpm, where $N_{rpm}$ is the number of rotations per minute of the tool. Torque measurements from the experiment were acquired from the FSW machine, and power outputs from the simulation were converted to torque values using Equation (6). The torque measurements from the simulation matched the experiment quite well, as seen in Figure 7. The peak torques were very similar, while the quasi-steady-state portion of the curve was somewhat underestimated in the simulation. Additionally, the steep increase in torque at 7 s when the tool shoulder engaged the workpiece was also captured by the simulation, although the simulation had a steeper slope. Torque is a good indicator of simulation accuracy, as it incorporates material behavior, as well as interface behavior, in terms of how friction resists the rotation of the tool.

### 3.2. Sensitivity of Temperatures to $h_{W/T}$

**Figure 8:**
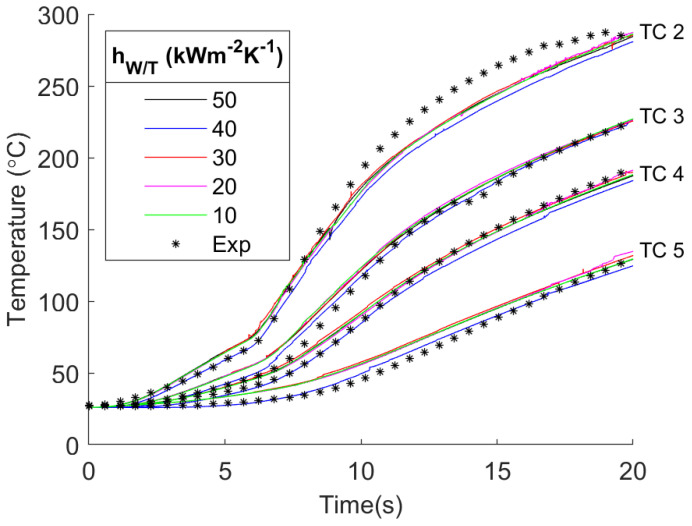
Results of different $h_{W/T}$ values for workpiece temperature plotted with the experimental values for comparison. For each value of $h_{W/T}$, there are four sets of lines plotted, one for each thermocouple location.

Having validated the model, the next step is to vary the values of $h_{W/T}$ while maintaining the tuned values of the friction coefficient. This is done to determine the effect of $h_{W/T}$ on the workpiece and tool temperatures. The values of $h_{W/T}$ used for the simulation were 10, 20, 30, 40, and 50 kW m${}^{-2}$ K${}^{-1}$, which were based on the higher range of values in the literature (Figure 2). Figure 8 shows how the different values of $h_{W/T}$ affected the temperature in the workpiece. As the value of $h_{W/T}$ increased, there was a negligible affect on the peak temperature (see Figure S8). The high *k* of the aluminum facilitated the transfer of heat from the interface to the boundaries of the workpiece at a sufficiently high rate, wherein changes in the $h_{W/T}$ led to minimal temperature changes in the workpiece. This result has the potential to be different for less thermally conductive materials such as stainless steel.

**Figure 9:**
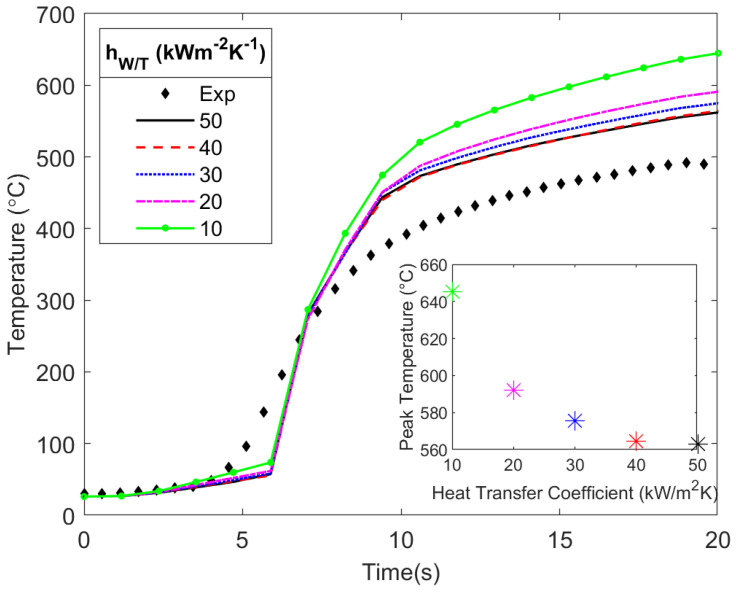
Effect of varying $h_{W/T}$ on tool temperature compared with the experimental results. A difference of about 140 °C was observed between simulations when the lowest and highest $h_{W/T}$ values were used.

In addition to the workpiece, the effect of $h_{W/T}$ on the tool temperature was also studied, where variations in the $h_{W/T}$ had a noticeable effect. Figure 9 shows that as the $h_{W/T}$ decreased, the temperature in the tool shoulder increased (see Figure S8). Also, as $h_{W/T}$ increased, the peak temperature in the tool decreased. The lower *k* of the H13 tool steel, relative to the aluminum workpiece, resulted in the buildup of heat close to the interface where the heat was generated. As $h_{W/T}$ decreased, less heat was conducted from the hotter tool to the cooler workpiece, thus further contributing to greater tool temperatures. This resulted in a higher temperature gradient, thus resulting in a higher temperature near the interface where the thermocouple sensor was located. There is likely a threshold value where further increasing $h_{W/T}$ no longer decreases the peak temperature in the tool, as could be seen with $h_{W/T}$ values equal to 40 and 50 kW m${}^{-2}$ K${}^{-1}$. The difference between 50 and 10 kW m${}^{-2}$ K${}^{-1}$, as shown in Figure 9, was around 140 °C, wherein the difference in peak temperature increased each time the $h_{W/T}$ decreased by 10 kW m${}^{-2}$ K${}^{-1}$. There was a difference of about 80 °C between the experimental tool temperature and the simulated temperature at $h_{W/T}$ = 40 to 50 kW m${}^{-2}$ K${}^{-1}$. This was the result of first matching the workpiece temperatures while having the secondary objective of matching the tool temperatures.

The discrepancy between the experiment and simulation in the case of tool temperature could be related to the sharing of frictional heat at the interface, which is partitioned based on the effusivities of the materials in contact. As such, the physical parameters of both the AA 6061 and the H13 materials were confirmed through several publicly available sources [29]. However, if these values are not accurate for the full range of temperatures that occurred during the plunge, then the sharing of heat could be a source of error in the simulation. The challenge of predicting tool temperatures in this case highlights why it is critical to validate a FSW welding model with measurements on both sides of the heat generation interface. It is relatively straightforward to match temperatures in either the tool or workpiece via model tuning, but it is far more difficult to match in both the tool and workpiece while also achieving a reasonable prediction for the spindle torque.

**Figure 10:**
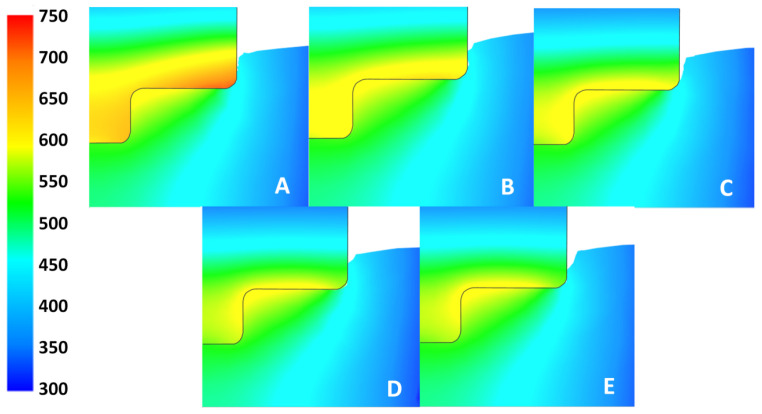
Thermal plots of the tool and workpiece (one-half of section view) for each simulated value of $h_{W/T}$ at 20 s. Plots (**A**–**E**) represent $h_{W/T}$ values of 10, 20, 30, 40, and 50 kW m${}^{-2}$ K${}^{-1}$, respectively. Temperature scale is in units of °C.

To visualize the effect of $h_{W/T}$ on both the tool and the workpiece simultaneously, Figure 10 compares the thermal gradients at the final time step of the simulation for all values of $h_{W/T}$. From Figure 8 and Figure 10, it is evident that the various $h_{W/T}$ values had a negligible effect on the workpiece temperature at the thermocouple sensor locations, as well as a negligible effect on the overall thermal gradient in the workpiece. However, Figure 8 and Figure 10 show that the $h_{W/T}$ did affect the tool temperature at the thermocouple sensor location, and the overall thermal gradient differed between the $h_{W/T}$ values. For $h_{W/T}$ = 10 kW m${}^{-2}$ K${}^{-1}$, more heat built up in the tool near the interface, because it was not able to conduct across the interface as easily as compared to $h_{W/T}$ = 50 kW m${}^{-2}$ K${}^{-1}$. Heat buildup did not occur in the workpiece because of the greater *k* and diffusivity of the aluminum workpiece.

## 4. Discussion

The current modeling results show that $h_{W/T}$ values less than 10 kW m${}^{-2}$ K${}^{-1}$ under-represent the heat transfer between the tool and the workpiece. Nakamura [20] employed a value of $h_{W/T}$ = 5 kW m${}^{-2}$ K${}^{-1}$, thereby showing a good match with tool temperature measurements, but no measurement in the workpiece was conducted, and, therefore, it is difficult to know how the model performed in predicting workpiece temperatures. Our results show that lower $h_{W/T}$ values provided tool temperatures that were well above those measured during the experiment. A greater value for $h_{W/T}$ is more likely to be the case, due to the high pressures and intimate contact between the tool and workpiece that is facilitated by intense shearing of the material, compared to other models that predict thermal contact conductance with similar pressure but under static conditions [30]. Also, a higher $h_{W/T}$ better matches the tool temperature as shown in Figure 9.

The results from this study are only of the transient plunge step of the FSW process. Therefore, comparisons between this and other works for steady-state models are not directly applicable. Steady-state models use a fixed friction coefficient for a transverse weld process, whereas a changing friction coefficient was used to adjust for the transient nature of the plunge process [2,20]. However, the current results can be compared to other works that have studied the plunge process [31,32,33]. Figure 10 agrees with work done by Yu et al. [34] showing similar thermal contours in the workpiece with the hottest location near the root of the tool and a similar thermal gradient moving out into the workpiece.

A possible source of error is the material properties of the model. These properties evolve with temperature and are only as accurate as the reference used for the simulation [29]. If these temperature-dependent properties are inaccurate, then the effusivity values for the workpiece and tool, used to partition the heat generated at the contact interface between them, would be affected. For an explanation of other model limitations, see Section S3.

Modeling of both the tool and workpiece should become a common practice when validating an FSW model. Doing so will help to increase accuracy of model predictions by ensuring that heat generation at the workpiece/tool interface and the interactions that take place across the boundary are correct. If only the tool or the workpiece is modeled and validated, then these interactions cannot be evaluated rigorously. Validating the model on both sides of the workpiece/tool contact interface serves to highlight where model predictions are lacking and points to a need for understanding nuanced phenomena like how the heat generated by the tool is partitioned across the interface. This work also points to the need for the independent determination of friction law parameters and heat transfer coefficients in order to render FSW models more robust and to improve their predictions against experiments.

## 5. Conclusions

Accurate modeling of FSW requires rigorous model validation, which should be done for both the tool and the workpiece. Model development requires tuning some parameters in order to match experimental results. The process parameters are highly coupled, which means parameter changes do not always have a predictable outcome. Normally, the workpiece temperatures are of the greatest interest because the resulting weld quality and properties are of value for FSW process development. For this reason, the model tuning simulations detailed here have been primarily focused on matching the workpiece temperatures. However, the model development also aimed to match the tool temperature, which led to using more accurate physical parameters to improve partitioning of heat at the tool/workpiece interface. Further work on developing friction law parameters that are independent of model tuning, via experiments, will lead to more predictable and robust models. At the present time, a validation approach where both the workpiece and tool results are matched with experiments via parameter tuning should lead to more accurate modeling than most prior efforts where partial validation has been typical.

Based on the results of the current work, the following conclusions are made:

1. A time-dependent friction coefficient provides accurate model predictions of the workpiece temperatures.
2. Decreasing the value of $h_{W/T}$ showed no noticeable change in the workpiece temperatures, as the high thermal conductivity of the AA 6061-T6 dissipated heat quickly. For a less thermally conductive workpiece, such as stainless steel or titanium, variations in $h_{W/T}$ would likely have a larger impact on the temperatures within the workpiece.
3. Decreasing the value of $h_{W/T}$ results in higher tool temperatures, as this lowers the amount of heat transferring across the contact interface to the workpiece.
4. The validation of model temperature predictions must be done on both sides of the workpiece/tool interface in order to achieve reasonable results. The model shows that the partitioning of the heat from the friction at this interface strongly influences temperature predictions and is dependent on accurate physical parameter data. Therefore, the typical validation approach of matching temperatures in just the tool, or just the workpiece, will not lead to a predictive model.

## Data Availability

The data are contained within the article or Supplementary Materials, or they can be requested.

## Supplementary Materials

The following supporting information can be downloaded at: https://www.mdpi.com/article/10.3390/ma17010198/s1, Table S1: Force and rotational speed of test groups. Table S2: Tensile properties of AA 6061-T6. Figure S1: (a) Test plate for plunging experiment, highlighting location and numbering of thermocouples, as well as where the tool will plunge. All distances in mm. (b) Tool dimensions showing location of thermocouples. All measurements in mm. Figure S2: AA 6061-T6 thermal conductivity and specific heat as a function of temperature, from [12,35]. Figure S3: The P1+/P1 element is piecewise linear in both velocity and pressure, enriched by a bubble function, b, which is interpolated over the four sub-tetrahedra defined by the centroid and the four vertices, ensuring the numerical stability of the element [36]. Figure S4: (a) Tool mesh. (b) Mesh for the workpiece before the simulation. (c) Mesh for the workpiece after the simulation runs to show the mesh refinement stays intact during the deformation of the simulation. Figure S5: Linear interpolation between the points shown above was used to define the values of α with respect to time. A sufficient number of points were chosen to represent the s-curve. Figure S6: Schematic of the relative sizes of the thermocouple sensor and the hole for positioning the sensor in the workpiece. The red area represents the possible error in positioning caused by the difference between hole diameter and width of the thermocouple sensor. A ceramic collar below the bead is used to secure the sensor with super glue but some error in positioning could still be possible. Figure S7: Plot of the simulated peak temperatures of each thermocouple for each value of *h_W/T_*. There is no significant change between peak temperatures as *h_W/T_* changes. Figure S8: A zoomed in view of Figure 7 to show more clearly the spread of tool temperature with changing *h_W/T_*. Refs. [12,29,35,36,37,38,39] are cited in the supplementary materials.
