# Supplementary material for: Effect of Workpiece/Tool Heat Transfer and Friction Coefficients on Accuracy of Simulated Temperatures and Torques in a Friction Stir Welding Plunge
Source: Materials (Basel). 2023 Dec 29;17(1):198. doi: 10.3390/ma17010198 (PMC10779896; doi:10.3390/ma17010198)
Supplement: Supplementary file 1 [file materials-17-00198-s001.zip › materials-2728275-supplementary.pdf]

S1 Methods

S1.1 Experimental procedure

All tests are force controlled with different tool rpm as seen in Table S1.

The AA 6061-T6 workpieces each have dimensions of $L \times W \times H$ = 152.4 × 152.4 × 12.7 mm, with type K thermocouples at locations shown in Fig. S1a. Placement of thermocouples directly under the tool in the workpiece is avoided to limit thermocouple movement during the plunge. The thermocouple locations are chosen to capture the thermal gradients that occur during a non-steady-state plunge, where temperatures vary spatially and temporally over a short period of time.

The tool has a shoulder diameter of 25.3 mm, body length from the top of tool to the shoulder of 90.7 mm, and a flat shoulder. The tool has an unthreaded straight pin with a diameter of 7.1 mm and a length of 4.5 mm. The tool is also prepared with type K thermocouples placed 1 mm above the tool surface in the center of the pin and in the shoulder, with locations shown in Fig. S1b.

Table S1: Force and rotational speed of test groups

Test #	kN	rpm
1	27	400
2	27	600
3	36	400
4	36	600

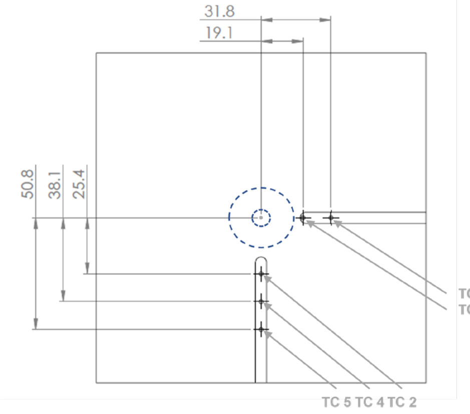

(a)

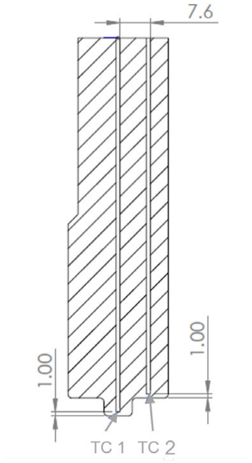

(b)

Figure S1: a) Test plate for plunging experiment, highlighting location and numbering of thermocouples, as well as where the tool will plunge. All distances in mm. b) Tool dimensions showing location of thermocouples. All measurements in mm.

S1.2 FSW Plunge Model

Material flow stresses and some material properties are provided by JMat-Pro [4] for AA 6061-T6. The temperature dependent material properties from Fig. S2 and the mechanical properties from Table S2 are used in the model. The nominal composition for AA6061-T6 is magnesium 0.8-1.2, silicon 0.4-0.8, copper 0.15-0.4, iron 0-0.7, chromium 0.04-0.35, zinc 0-0.25, and titanium 0-0.15 (in wt.%). The material properties for H13 steel are ρ = 7850 kg m^{-3}, c_p = 460 J kg^{-1} K^{-1}, and k = 24.3 W m^{-1} K^{-1}.

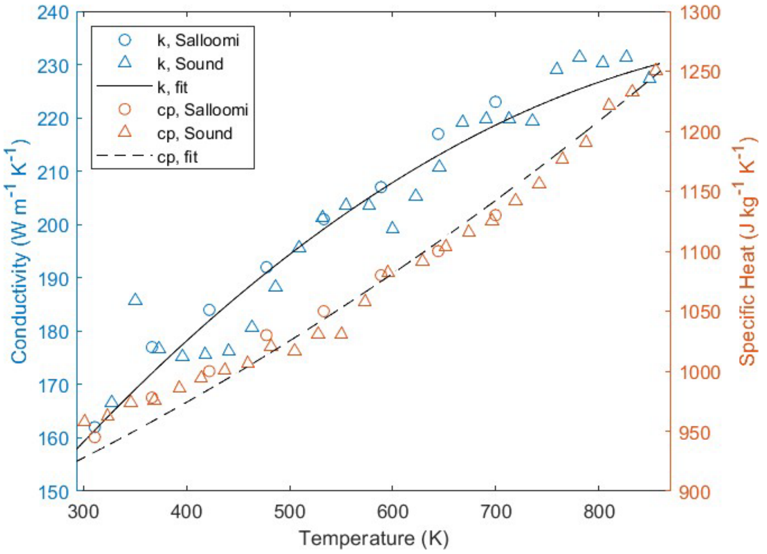

Figure S2: AA 6061-T6 thermal conductivity and specific heat as a function of temperature, from [1], [2].

Table S2: Tensile properties of AA 6061-T6

Yield Strength, MPa	Ultimate Tensile Strength, MPa	Total Elongation, %
276	27	400

The finite element discretization is based on an enhanced (P1 +/P1) 4-node tetrahedron element, as shown in Fig. S3. Temperatures are also interpolated using a piecewise linear function. The tool and workpiece meshes are shown in Fig. S4, where refinement was used in areas where large thermal and deformation gradients were expected, and elsewhere larger elements were employed to reduce computation time.

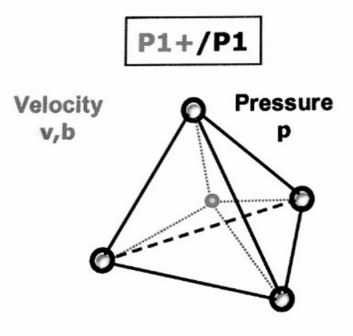

Figure S3: The P1+/P1 element is piecewise linear in both velocity and pressure, enriched by a bubble function, b, which is interpolated over the four sub-tetrahedra defined by the centroid and the four vertices, ensuring the numerical stability of the element [3].

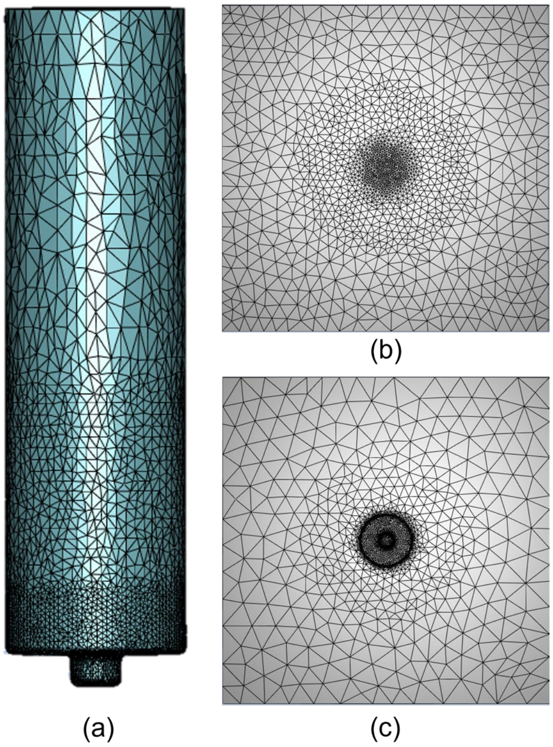

Figure S4: (a) Tool mesh. (b) Mesh for the workpiece before the simulation. (c) Mesh for the workpiece after the simulation runs to show the mesh refinement stays intact during the deformation of the simulation.

S1.3 Model Justification

To determine the validity of the simplifications, a transient heat transfer analysis based on tool properties, tool length, and length of the experiment will be discussed. The tool holder in the experiment surrounded the tool, leaving 29 mm from the shoulder of the tool exposed. The plunge lasted 20 seconds, as measured by the TCs. To determine if the tool could be treated as a semi-infinite medium for heat transfer, the Fourier number is calculated to determine if it is sufficiently low ($Fo = \frac{\alpha_{th} t}{L^2} < 0.2$ [5], where α_{th} is the thermal diffusivity of the tool, t is time, and L is the length of the exposed portion of the tool). The calculated Fo equals 0.16, which indicates that a semi-infinite assumption is valid, the heat transfer in the tool is not affected by the boundary condition between the tool and tool holder. This means that the heat generated at the tool/workpiece interface during the time of the experiment should not conduct to where the tool holder would start to affect the heat transfer. For longer experiments, the model should be modified to include the holder extending down the tool and a non-adiabatic condition should be imposed between them.

S2 Analysis/Results

The process of tuning the model consists of adjusting the friction coefficient, a, which is often modeled as a constant for the entire duration of a simulation. In the current study, the approach is to vary the friction coefficient as a function of time in order to better match the temperature profiles from the experiments. Chiumenti states that the friction coefficient is a non-linear function of the temperature and relative slip velocity making calibration difficult [6]. Fig. S5 shows how the friction coefficient was chosen to vary with time following an s-curve approach, as seen in prior work [7]. This friction curve is generated using an error function centered on the average of the maximum friction coefficient, before 7 seconds, and the minimum, after 11 seconds. The coefficient changes as the shoulder of the tool contacts the workpiece at around 7 seconds. Using a constant friction coefficient results in either extreme temperature values after the shoulder has plunged into the workpiece with mismatching slopes or inadequate heat generation and much lower temperatures for all times at the sensor locations. Thus, a greater coefficient that transitions to a lower value as the shoulder contacts

the workpiece results in the best matching of the experimental temperature profiles. As the temperature of the aluminum workpiece increases, properties like the yield strength and Young's modulus drop to about 25% of their room temperature values [1]. As a result, less friction occurs between the tool and workpiece as the temperature increases because the local shear stress that must be overcome to rotate the tool decreases. For the entire process of tuning, the heat transfer coefficient, $h_{W/T}$, was kept constant at 40 kW m^{-2} K^{-1}.

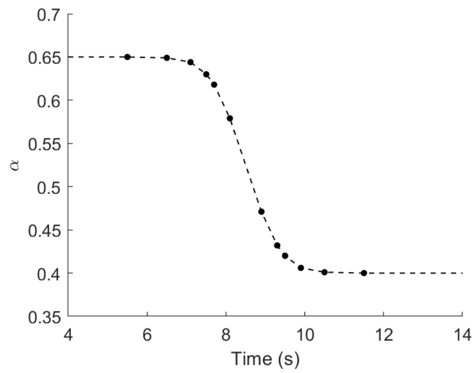

Figure S5: Linear interpolation between the points shown above was used to define the values of α with respect to time. A sufficient number of points were chosen to represent the s-curve.

An iterative approach is adopted for tuning of the friction coefficient. The specific friction law used for tuning is Norton's viscoplastic law. In this law, α is the coefficient of friction and the value that is tuned in the simulations. The initial and final values of α are subjected to the iterative tuning process, as the values that make up the curve between are auto generated using an error function, as discussed above. In addition to effects on the slope, a high initial value would cause the simulation temperatures to exceed the experimental values regardless of the final α value. For a fixed friction coefficient, matching the experiment during the first 7 seconds resulted in higher temperatures during and after the shoulder plunged into the workpiece. This is why the s-curve begins as the shoulder contacts the workpiece around 7 seconds. After tuning, the initial α value is 0.65 and the final value was 0.4, see Fig. S5 for intermediate values.

Numerical sensors are defined in the simulation workpiece at the same

locations as thermocouples in the experimental workpiece. Due to the workpiece hole tolerances where the thermocouples are located in the experiments, there is a possibility that a thermocouple could move from the center of the hole. To account for this possible source of error, the thermocouple width is measured and compared with the hole diameter to determine the possible variation in thermocouple position. Numerical sensors are introduced into the simulation at the center location as well as at distances that are possible within the hole dimensions where the thermocouple is placed. This accounts for some possible error in thermocouple positioning for the purpose of achieving a temperature match with the experiments, and is done because temperature gradients are very high in these areas of the workpiece for a short duration plunge experiment. Fig. S6 illustrates this point with a diagram of a thermocouple position in the workpiece. A match is achieved when the simulation results show similar values and slopes for temperature versus time plots when compared against the experimental values. Matching of the workpiece thermocouple temperatures is done with $h_{W/T}$ = 40 kW m^{-2} K^{-1} and a time dependent friction coefficient. Salloomi showed that a varying friction coefficient accounts for a changing sticking/slipping condition that exists between the tool and workpiece as temperature increases [1]. All other parameters match those listed in Section 2.2.1.

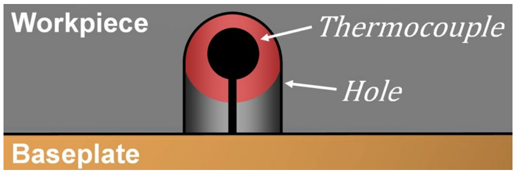

Figure S6: Schematic of the relative sizes of the thermocouple sensor and the hole for positioning the sensor in the workpiece. The red area represents the possible error in positioning caused by the difference between hole diameter and width of the thermocouple sensor. A ceramic collar below the bead is used to secure the sensor with super glue but some error in positioning could still be possible.

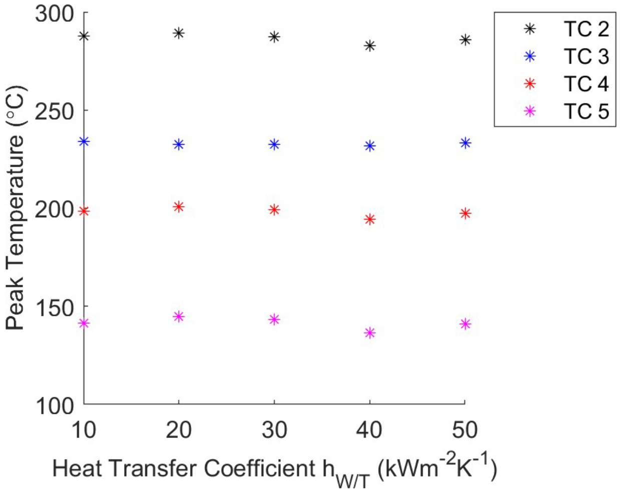

Figure S7: Plot of the simulated peak temperatures of each thermocouple for each value of $h_{W/T}$. There is no significant change between peak temperatures as $h_{W/T}$ changes.

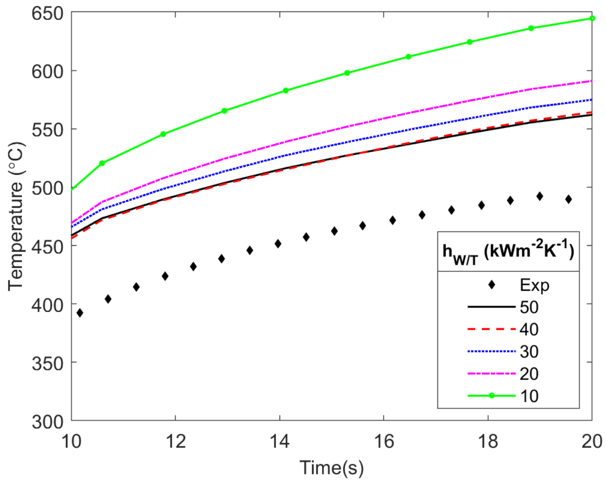

Figure S8: A zoomed in view of Figure 7 to show more clearly the spread of tool temperature with changing $h_{W/T}$.

S3 Discussion

Another previously mentioned limitation of the model is the boundary condition of the tool holder. For the 20 second plunge, the adiabatic boundary condition at the top of the tool does not materially affect the heat transfer within the tool. This is because the heat generated at the tool/workpiece interface does not have sufficient time to conduct through the tool to the holder. However, for a simulation longer than 20 seconds, the tool/holder boundary condition should play a role in the heat transfer because when $Fo > 0.2$, the tool can no longer be treated as semi-infinite. Also, the simple model geometry of the holder would need to be changed from what is shown in Fig. 5 to better match the FSW machine tool holder shown in Fig. 3. Using a more complete boundary on the tool shank could result in a marginal improvement in the current results.

A clear limitation of this model and all FSW models is that the friction coefficient is a fitting parameter. To simulate other tool geometries, process parameters, or materials, a new fit for the friction coefficient would need to be done for each case, coupled with selecting an appropriate heat transfer coefficient. Future work focused on experimentally measuring the $h_{W/T}$ coefficient would decouple the friction coefficient and heat transfer, enabling the independent tuning of frictional parameters within models. But the current results show that an S-shaped curve for the friction coefficient (Fig. S5), as a function of time, is helpful in tuning the interface heat generation to predict the experimental temperatures in workpiece and tool.

References

[1] Salloomi KN. Fully coupled thermomechanical simulation of friction stir welding of aluminum 6061-T6 alloy T-joint. Journal of Manufacturing Processes. 2019 5;45:746-54.

[2] Soundararajan V, Zekovic S, Kovacevic R. Thermo-mechanical model with adaptive boundary conditions for friction stir welding of Al 6061. International Journal of Machine Tools and Manufacture. 2005 3;45:1577-87.

[3] Pichelin E, Coupez T. Finite element solution of the 3D mold filling problem for viscous incompressible fluid. Computer Methods in Applied Mechanics and Engineering. 1998;163:359-71.

[4] Ltd SS. JMatPro; 2014.

[5] Bergman TL, Lavine AS, Incropera FP, Dewitt DP. Fundamentals of Heat and Mass Transfer. John Wiley & Sons; 2011.

[6] Chiumenti M, Cervera M, de Saracibar CA, Dialami N. Numerical modeling of friction stir welding processes. Computer Methods in Applied Mechanics and Engineering. 2013 1;254:353-69.

[7] Meyghani B, Awang M, Emamian S, Khalid NM. Developing a Finite Element Model for Thermal Analysis of Friction Stir Welding by Calculating Temperature Dependent Friction Coefficient. Springer; 2017. p. 107-26.
